# Marine derived biosurfactants: a vast potential future resource

**DOI:** 10.1007/s10529-018-2602-8

**Published:** 2018-08-25

**Authors:** Lakshmi Tripathi, Victor U. Irorere, Roger Marchant, Ibrahim M. Banat

**Affiliations:** School of Biomedical Sciences, Faculty of Life and Health Sciences, Ulster University, Coleraine, BT52 1SA UK

**Keywords:** Surface-active, Marine bacteria, Biosurfactant, Emulsifier, Bioremediation, Biodegradable

## Abstract

Surfactants and emulsifiers are surface-active compounds (SACs) which play an important role in various industrial processes and products due to their interfacial properties. Many of the chemical surfactants in use today are produced from non-renewable petrochemical feedstocks, while biosurfactants (BS) produced by microorganisms from renewable feedstocks are considered viable alternatives to petroleum based surfactants, due to their biodegradability and eco-friendly nature. However, some well-characterised BS producers are pathogenic and therefore, not appropriate for scaled-up production. Marine-derived BS have been found to be produced by non-pathogenic organisms making them attractive possibilities for exploitation in commercial products. Additionally, BS produced from marine bacteria may show excellent activity at extreme conditions (temperature, pH and salinity). Despite being non-pathogenic, marine-derived BS have not been exploited commercially due to their low yields, insufficient structural elucidation and uncharacterised genes. Therefore, optimization of BS production conditions in marine bacteria, characterization of the compounds produced as well as the genes involved in the biosynthesis are necessary to improve cost-efficiency and realise the industrial demands of SACs.

## Surfactant industry and problem identification

Surfactants are utilized in various bulk commercial products particularly in personal care products and household cleaners. To fulfil their worldwide demand, millions of tonnes of surfactants are annually produced from non-renewable sources such as petrochemicals. Consumption of surfactants is probably greater than 13 million tonnes per year worldwide (Marchant and Banat 2012a). The most widely used synthetic surfactants such as alkyl benzene sulfonates (ABS), are not readily biodegradable thus causing adverse effects on the environment. U.S. detergent manufacturers therefore, replaced ABS with linear alkylbenzene sulfonate (LAS) which showed no adverse impact on the environment and had the same cleaning characteristics (Cowan-Ellsberry et al. 2014). However, diminishing petrochemical stocks have created a drive towards identification of novel renewable bioresources for efficient surfactant production (Foley et al. 2012). In recent years researchers have been involved in continued environmental research to source replacements for synthetic surfactants and other bioactives from the marine environment (Kalogerakis et al. 2015). Surface-active compounds (SACs) produced by microorganisms offer an ideal sustainable substitute for petrochemical based surfactants. Biologically produced SACs have numerous potential applications in a wide variety of industrial sectors, due to their synthesis from waste and renewable substrates such as, hydrocarbon wastes, crude oil and vegetable oils in addition to being bio-compatible, non-toxic and biodegradable (Banat et al. 2010). They are usually classified based on their molecular weight, those surfactant molecules with low molecular weights are known as biosurfactants (BS) whilst those with high molecular weights are known as bioemulsifiers (BE).

BS are further categorized based upon their molecular structure e.g., glycolipids, lipopeptides, phospholipids, lipoprotein, fatty acids and polymeric BS. They also have a range of different properties such as, surface tension reduction, emulsification, foaming and wetting (Banat et al. 2014; Marchant and Banat 2012a). With over 5.73 million tonnes of oil spills in the oceans worldwide between 1970 and 2016 (ITOPF 2017), there is an urgent demand for novel bioactive compounds for bioremediation. BS naturally play a major role in bioremediation following oil spills and act as efficient dispersing agents facilitating microbial biodegradation (De Almeida et al. 2016; Franzetti et al. 2010). Despite their versatile properties, a higher production cost compared to chemical surfactants and the pathogenicity of some BS producing strains, remain a major obstacles for their large-scale production (Irorere et al. 2018) and the search for non-pathogenic strains remain an important research area (Elshikh et al. 2017; Funston et al. 2016). Therefore, an important question to address before they can achieve widespread application is “can we develop a cost-effective and ecologically benign process for BS production?”

## Surfactant production in marine bacteria

Surfactants with diverse properties and low production costs are required to increase the applications of natural SACs, which gives greater incentive to develop surfactants of biological origin produced by microorganisms. Marine microorganisms are ubiquitous in the marine environment as well as extreme environments. The oceans have a relatively narrow range of pH, salinity and temperature while areas such as the volcanic vents face extreme conditions. These microorganisms are known to be metabolically and physiologically adapted to survive under extreme temperature, pressure, pH and salinity conditions (Das et al. 2010; Thavasi et al. 2014). For example, members of the *Alcanivorax* genus survive at low to mild hydrostatic pressure in hydrocarbon contaminated environments. The strains *A. borkumensis* SK2 and *A. dieselolei* KS 293 have developed different strategies to cope with environmental stress under high pressure. While the respiration and cell integrity is not affected in KS 293 at mild hydrostatic pressure, SK2 activates the production of the osmolyte ectoine to cope with hydrostatic pressure (Scoma et al. 2016a, b). Marine bacteria secrete large molecules known as exopolysaccharides (EPS) consisting of proteins, polysaccharides, lipids, nucleic acids and uronic acids. EPS enhances the survival of microbial cells under changing environmental conditions through various mechanisms such as, biofilm formation, enhancing substrate adhesion, protection against limited nutrient availability, detoxification of metals and the presence of antibiotics (Harimawan and Ting 2016; Pal and Paul 2008).

Some microorganisms specifically produce amphiphilic EPS, particularly BS as a mechanism to increase the bioavailability of hydrophobic substrates such as hydrocarbons, these BS enhance the growth of indigenous bacteria capable of degrading aliphatic and aromatic hydrocarbons. BS produced from marine bacteria can facilitate hydrocarbon dispersion, degradation, emulsification and bioavailability (Das et al. 2010; Mapelli and Scoma 2017). BS from cold-adapted marine microorganisms or psychrophilic organisms can work efficiently at cold and freezing temperatures, and are therefore suitable in laundry detergent formulations where low temperature washing conditions have become a priority for energy conservation (Perfumo et al. 2018; Marchant and Banat 2012b). The potential uses of BS are further improved by their low-toxicity, meaning they are applicable for large-scale industrial production and subsequent environmental disposal where they can be readily biodegraded (Irorere et al. 2017; Uzoigwe et al. 2015). Hence marine bacteria offer an excellent opportunity for the discovery of new SAC molecules with distinctive properties. Although highly attractive, the biosynthesis of BS from marine organisms has largely been overlooked. The mechanism of their regulation during synthesis is also not fully understood adding further difficulties to the process for their production. Several approaches are required before the widespread application of marine-derived BS can be achieved: (i) Isolation and identification of novel, non-pathogenic marine BS producing bacteria (ii) Optimization of culture conditions to achieve sufficient yields of BS and (iii) Characterization of genes involved in BS production from marine organisms. These will allow the use of marine strains in large scale BS production processes while improving yield and cost-efficiency of BS production.

### Isolation of BS producing marine strains

Much of the research on BS is focused on soil isolates, including the *Pseudomonas* and *Bacillus* groups, however, BS production by marine microorganisms is an area relatively unexplored since it is still considered that marine microorganisms are difficult to culture in the laboratory (Stein et al. 1996; Walsh and Duffy 2013). The global demand for BS has led to a number of academic research groups and manufacturers searching underexploited sources such as bacteria isolated from the marine environment. Recent reports have shown the successful isolation and culture of diverse BS producing microorganisms from marine habitats (Thavasi et al. 2009). BS production is induced in most marine bacteria in the hydrocarbon polluted environment. Marine bacteria increase hydrocarbon bioavailability by the production of BS which shows surface/emulsification activities and facilitates hydrocarbon degradation (Thavasi et al. 2011). The marine oil spill from deep water horizon in the Gulf of Mexico caused large volumes of oil spill into the water. Several BS producing marine bacteria became predominant following the spill, including *Alteromonas*, *Halomonas*, *Alcanivorax*, *Colwellia*, *Cycloclasticus* and *Pseudoalteromonas* (Mapelli and Scoma 2017). A number of other bacteria producing BS including, *Pseudomonas*, *Bacillus*, and *Acinetobacter* were reported in oil contaminated waters worldwide (Gerard et al. 1997; Hentati et al. 2016; Ortega-de la Rosa et al. 2018), although these species are not necessarily restricted to the marine environment.

The populations of marine bacteria can be increased by the addition of inorganic nutrients which allows them to use hydrocarbons as a carbon and energy source. *A. borkumensis* is found in low numbers in unpolluted environments but becomes the dominant microbe in hydrocarbon polluted ocean and coastal waters. The growth and multiplication of *Alcanivorax* in oil treated sea water increased up to 91% within 1–2 weeks of nutrient supplementation (Syutsubo et al. 2001). It was observed that addition of inorganic nutrients improved the dynamics of the bacterial community (Roling et al. 2002). Providing suitable conditions for the hydrocarbon utilization can be an efficient method to achieve growth and multiplication of these organisms. In microcosm and field biodegradation experiments, bacteria with 16S rRNA sequences related to *A. borkumensis* and *Pseudomonas stutzeri* increased when supplemented with oil and inorganic nutrients (Roling et al. 2004).

Considering that the marine environment contains diverse marine microbes numbering approximately 3 × 10^28^ bacteria (Copley 2002), metagenomic-based approaches can be applied for the discovery of novel marine microbial derived BS (Kennedy et al. 2011). Metagenomic analyses involve both sequence-based and function-based strategies. Function-based strategies are used to screen metagenomics libraries constructed from marine ecosystems which typically involves using *Escherichia coli* as the heterologous host and the subsequent screening of this library. Sequence-based analyses, involves the identification of genes based upon homology with well characterized genes that are found in sequence databases (e.g., BLAST, MEGAN, KEGG). However, the major bottleneck for identifying genes from metagenomic sources is the identification of coding regions that do not have homology in the sequence databases. Additionally, the metagenomic approach requires a deeper knowledge of the diversity of BS molecules produced and the underlying genetic systems than we have at present.

### Culture conditions for production of BS from marine bacteria

The quality and productivity of BS are determined by the bacterial strain and their growth conditions. The optimization of growth conditions is important for the maximum production of BS. The composition and productivity of BS are also influenced by carbon source, pH, temperature, salinity, nitrogen and agitation amongst other factors. Marine microorganisms which require salts for growth are referred to as halophiles. Many marine organisms such as moderate halophiles require 3–15% (w/v) NaCl for optimal growth while extreme halophiles grow optimally at 25% (w/v) NaCl (Margesin and Schinner 2001). *Halomonas* are known to produce different types of glycolipids and glycoproteins with superior emulsifying activity than commercial emulsifiers. These halophilic bacteria may play a vital role for the production of surfactants and emulsifiers in oil-polluted saline environments. The growth of bacteria is influenced by salt concentration which also affects BS production. The growth of halophilic bacteria at high salt concentration can reduce the contamination risks which can significantly reduce upstream fermentation costs of BS production (Pepi et al. 2005; Tan et al. 2011). However, for large scale industrial production growth media containing high salt concentration would be regarded as undesirable due to the corrosive effects it would have on the production plant infra structure.

Different carbon sources in the growth medium influence the composition of BS production. Carbon sources studied for BS production include crude oil, diesel, glucose, sucrose and glycerol. Several studies show that marine bacteria can utilize hydrocarbons as substrate and produce BS, therefore the ability of hydrocarbon degrading marine bacteria to produce BS could be utilized in the bioremediation of hydrocarbon-contaminated environments. *Halomonas* sp. strain C2SS100 degraded hydrocarbons and produced BS at high salinity (Mnif et al. 2009), while *Brevibacterium luteolum* synthesized BS using mineral oil as a carbon source (Vilela et al. 2014). A *Brevibacillus* strain degraded phenanthrene to produce BS (Reddy et al. 2010) and *Alteromonas* sp. 17 degraded hydrocarbons and produced BS using long chain alkane eicosane (Al-Mallah et al. 1990). Glycolipopeptide BS was also produced by *Corynebacterium kutscheri* using cheaper carbon sources like waste motor lubricant oil and peanut oil cake (Thavasi et al. 2007). It is important to bear in mind that for large-scale commercial production of BS the cost of the growth medium may only constitute a smaller contribution to the total production costs when the energy required to run the fermentation for a prolonged period is taken into account and since many renewable plant oils are relatively cheap, readily available and of consistent composition. Therefore, the obsessive search for ‘waste’ materials as substrates towards achieving cost effective processes may be unjustified.

Nitrogen is vital for microbial growth and BS production. The type and concentration of the nitrogen source plays an essential role in the optimization of BS production (Davis et al. 1999). Different nitrogen sources could be used for the production of BS including, yeast extract, urea, peptone, ammonium sulphate, ammonium nitrate and sodium nitrate. Yeast extract was the best nitrogen source for the production of the BS in the marine *Streptomyces* species B3 (Khopade et al. 2012b). Sodium nitrate and yeast extract were preferred nitrogen sources for BS production by marine *B. subtilis* N3-4P (Zhu et al. 2016) while the maximum BS production was observed with phenyl alanine as the nitrogen source in marine *Nocardiopsis* B4 (Khopade et al. 2012a).

Another important parameter is temperature that greatly influences cell growth and BS production. BS from thermophilic microorganisms are industrially preferred due to their thermostability at temperatures above 40 °C, however BS produced from mesophiles also have high levels of thermo-stability and psychrophilic marine bacteria capable of producing BS have potential applications for bioremediation in cold environments. The hydrocarbon-degrading bacterium, *Rhodococcus* sp. obtained from the Norwegian coastline produced BS during cultivation at 20 °C with kerosene, *n*-hexadecane or rapeseed oil as a carbon source (Dang et al. 2016). The results reported above clearly indicate that conditions for growth and BS production are often organism specific and each organism isolated will need to be fully investigated to optimise both medium and production conditions.

### Characterization of BS production in marine bacteria

The identification of possible genes involved during BS synthesis is necessary in order to understand BS synthesis and develop robust BS producing strains with high production capacity. The most well-characterized low-molecular weight BS is rhamnolipid, produced by several species of *Pseudomonads* and *Burkholderia*. The genes *rhlA*, *rhlB* and *rhlC*, which are responsible for the biosynthesis of rhamnolipids have been found in *P. aeruginosa* as well as non-pathogenic *B. thailandensis*. These three genes are localized within a single gene cluster in *Burkholderia* while, the *rhlC* gene is located at two different, remote position within the *P. aeruginosa* genome (Dubeau et al. 2009; Perfumo et al. 2013). Recently, homologues to *P. aeruginosa* rhamnolipid genes *rhlA* and *rhlB* were identified in a non-pathogenic marine *Pseudomonas* species MCTG214(3b1) (Twigg et al. 2018). Most of the genetic studies of BS production are limited to well-characterised BS molecules, but the expression of genes involved in BS synthesis is not well studied in marine bacteria.

The genes and regulatory pathways are not necessarily identical in different BS producers. Different species can produce a BS with totally different chemical structure and even small variations in the congener composition of a surfactant can greatly affect its functional property. To fully understand how SAC synthesis is regulated in these marine strains, it is important to characterise the chemical structure and necessary genes required for BS synthesis. The chemical composition of the SAC molecules can be determined by electrospray ionization mass spectrometry (ESI–MS), high performance liquid chromatography mass spectrometry (HPLC–MS) and nuclear magnetic resonance (NMR) techniques (Smyth et al. 2010).

Few reports have been published regarding BS synthesis during hydrocarbon degradation in marine bacteria. It was reported that *P. aeruginosa* JP-11 isolated from the marine environment utilized 98.8% ± 2.3% of biphenyl within 72 h from contaminated sites. Although *P. aeruginosa* cannot be considered a true marine bacterium, it is a common organism isolated from the marine environment. The production of BS was confirmed by the expression of the rhamnolipid synthesizing genes *rhlAB* (Chakraborty and Das 2016). *Bacillus* species are known to produce BS such as lichenysin, surfactin, fengycin, pumilacidin, iturin and bacillomycin (Vater et al. 2002). BS production was seen during anthracene degradation by a marine alkaliphile *Bacillus licheniformis* (MTCC 5514). The strain degraded > 95% of 300 ppm anthracene and showed tolerance up to 500 ppm of anthracene concentration. The gene involved in the BS lichenysin production was *licA3*, followed by degradation through the catabolic degradative enzyme, catechol 2,3 dioxygenase(C23O) (Swaathy et al. 2014).

*Acinetobacter* species are also known to produce high-molecular weight emulsifiers (Ortega-de la Rosa et al. 2018). In *Acinetobacter lwoffii* RAG-1, the genes encoding the biosynthesis of emulsan (a polysaccharide BE) were reported to be clustered within a 27-kbp region termed, wee cluster (Nakar and Gutnick 2001). The bioemulsan alasan produced by *A. radioresistens* is a complex mixture of anionic polysaccharides and protein. The *alnA* gene which codes for the surface-active protein of alasan was cloned, sequenced, and expressed in *E. coli*. Significant sequence similarity (21%) between the recombinant emulsifier protein AlnA of *A. radioresistens* and OmpA of *E. coli* was seen. However, no emulsifying or hydrocarbon solubilizing activities have been observed with *E. coli* OmpA (Toren et al. 2002). It has also been reported that the marine hydrocarbonoclastic bacterium *Alcanivorax borkumensis* synthesizes glycolipids for hydrocarbon degradation (Abraham et al. 1998; Yakimov et al. 1998). The genome sequence of *A. borkumensis* SK2 revealed its capacity for BS production. The glycosyltransferase (ABO_1783) similar to RhlB from *P. aeruginosa* and glycosyltransferase protein family 9 (ABO_2215) were found to be potentially involved in BS production. The *A. borkumensis* SK2 genome encodes other proteins involved in emulsifier production namely, OmpA (ABO_0822), OprG/OmpW (ABO_1922) and OmpH (ABO_1152) (Schneiker et al. 2006). Similarly, genes involved in BS production were reported in the marine bacterium *Achromobacter* sp. HZ01. The genome of strain HZ01 harbours OmpH (gene_1336) and OmpA (gene_2469) which are both related to emulsifier production (Hong et al. 2017). Similar genes involved in biosynthesis of BS were found in the genome of *Cobetia* sp. MM1IDA2H-1 (Ibacache-Quiroga et al. 2017). A significant problem in using genetic information from one organism to another is the lack of sequence homology between genes which may lead to the production of similar products. The rhamnolipid production genes in *P. aeruginosa* PAO1 and *B. thailandensis* have only about 50% sequence homology (Dubeau et al. 2009). While in a marine *Pseudomonas* species MCTG214(3b1), unrelated to *P. aeruginosa*, which produces di-rhamnolipids identical in structure to *P. aeruginosa,* the *rhlA* and *rhlB* genes have very high sequence homology while it has so far been impossible to find an *rhlC* homologue which indicates the presence of second novel rhamnosyltransferase (Twigg et al. 2018). This suggests that it will be necessary to search for specific domains within the genes rather than whole gene sequences when investigating such capabilities.

## Marine-derived BS in bioremediation

The release of petroleum hydrocarbons in marine environments due to oil spill and chronic pollution is a serious major concern. Chemical dispersants are effectively utilized worldwide to minimize oil spill damage. Dispersants are mixtures of one or more surfactants and solvents that enhance dispersion of oil into droplets leading to increased mobility and bioavailability of hydrocarbons. The dispersed oils are solubilized in water and degraded by microorganisms. However, chemical dispersants are toxic to aquatic species and replacing them with biological non-toxic alternatives would be very advantageous and highly sought after. Various marine γ-proteobacteria are known to secrete cell surface amphiphilic substances (BS or BE) that allow the solubilization of aromatic hydrocarbons. During growth on hydrocarbons, microbial cells attach to oil droplets by secreting BS to increase the bioavailability of hydrocarbons. BS production by these bacteria increases dispersion of hydrocarbons thereby enhancing their degradation by non-BS-producing microorganisms (McGenity et al. 2012; Perfumo et al. 2010).

Marine microorganisms such as *Halomonas*, *Marinobacter*, *Myroides* as well as the tropical marine yeast *Yarrowia lipolytica* can play an important role in the ultimate removal of hydrocarbon compounds from contaminated sites by the production of BE. Due to their diverse structural and functional property, these BE have potential applications in bioremediation processes (Table 1). The *Halomonas* sp. participate in the removal of spilled oil by synthesizing surface-active emulsifiers. Glycolipid molecules produced by *Halomonas* sp. on their cell surface enhance the solubility of hydrocarbon, and thus increase their bioavailability for degradation. The emulsifying BS produced by *Halomonas* sp. could be used for enhanced oil recovery processes in extreme environments (Dhasayan et al. 2014), since the emulsifier produced by this bacterium at low temperature could enhance the bioavailability of hydrocarbons in cold environments (Pepi et al. 2005).

**Table 1:** Summary of biosurfactant/bioemulsifier suggested for bioremediation applications produced by different marine microorganisms

Organism	Organism identification method(s)	Biosurfactant type	Biosurfactant property	BS/BE yield	Characterisation method	Reference
*Halomonas* sp. MB-30	16S rRNA	Glycolipid	MEOR	NA	FTIR, NMR	Dhasayan et al. (2014)
*Brevibacterium luteolum*	16S rRNA	Lipopeptide	Bioremediation	NA	FTIR	Vilela et al. (2014)
*Myroides* sp. SM1	16S rRNA	l-ornithine lipid	Emulsify crude oil	2.6 g/10 l	MS, NMR, FTIR, GC–MS	Maneerat et al. (2006)
*Acinetobacter calcoaceticus* RAG-1	Enrichment culture technique	Emulsan: heteropolysaccharide protein	Bioremediation, MEOR	1.5 g/27 l (heptane extraction), 2.1 g/10 l (ammonium sulphate precipitation)	IR, NMR, GLC	Belsky et al. (1979), Reisfeld et al. (1972), Rosenberg et al. (1979)
*Marinobacter* sp.	Identified according to Bergey’s manual of systematic bacteriology	Carbohydrates: lipids complex	Emulsify HCs	NA	TLC, GC	Al-Mallah et al. (1990)
*Marinobacter* sp.	16S rRNA	Phospholipopeptide	Biodispersant	NA	FTIR	Raddadi et al. (2017)
*Yarrowia lipolytica*	NA	Glycoprotein	Water-in-oil emulsions with HCs and with PFCs	NA	FTIR, Raman and NMR	Amaral et al. (2006)
*B. stratosphericus* FLU5	16S rRNA	NA	Bioremediation	NA	NA	Hentati et al. (2016)
*Achromobacter* sp. HZ01	16S rRNA	Lipopeptide	Emulsify HCs	6.8 g/l	FTIR, GC–MS, HPLC-LTQ-Orbitrap MS	Deng et al. (2016)
*Nocardiopsis* VITSISB (KC958579)	16S rRNA	Rhamnolipid	Bioremediation of oil spill	NA	FTIR, GC–MS	Roy et al. (2015)

*MEOR* microbial enhanced oil recovery, *NA* not available, *TLC* thin-layer chromatography, *FTIR* Fourier transform infrared spectrometry, *NMR* nuclear magnetic resonance, *MS* mass spectrometry, *IR* infrared, *HPLC*-*LTQ*-*orbitrap* high performance liquid chromatography coupled with linear ion trap-orbitrap mass spectrometry, *GC* gas chromatography, *GLC* gas liquid chromatography, *HCs* hydrocarbons, *PFC* perfluorocarbons

Ornithine lipids, another type of BE produced by *Myroides* sp. SM1 have strong emulsification ability for crude oil and stability in a wide range of temperatures and pH. It was reported to show better surface activity than synthetic detergents and surfactin (Maneerat et al. 2006). Emulsan, the most powerful emulsion stabilizer produced by *Acinetobacter calcoaceticus* RAG-1 was reported to have potential applications in microbial enhanced oil recovery (MEOR) and cleaning oil spills (Belsky et al. 1979; Rosenberg et al. 1979). Other biosurfactants have also been reported to enhance oil/hydrocarbon bioremediation activity (Banat et al. 2000; Franzetti et al. 2011) The bioemulsifier produced by *Alteromonas* sp. 17 (now known as *Marinobacter*) could also be used for the efficient degradation of hydrocarbons (Al-Mallah et al. 1990). In another report, a *Marinobacter* species produced a phospholipopeptide class of BS capable of emulsification. The emulsions showed low ecotoxicity and were able to disperse crude oil in artificial marine water suggesting their application for bioremediation purposes (Raddadi et al. 2017). The glycolipid BS produced from *Marinobacter hydrocarbonoclasticus* strain SdK644 showed twofold greater solubilisation of crude oil than tween 80 hence showing potential in marine based bioremediation (Zenati et al. 2018). Another emulsifier “Yansan” produced by *Yarrowia lipolytica*, an aerobic yeast showed high emulsification activity and stability over a pH range of 3–9 and potential applications in the formulation of perfluorocarbon (PFC) based emulsions and degradation of hydrocarbons (Amaral et al. 2006). Biosurfactants produced by *Candida lipolytica* yeast strain was also used formulating a commercial related product for oil bioremediation (Santos et al. 2017).

## Marine-derived BS as antimicrobial and therapeutic agents

The indiscriminate use of antibiotics leading to drug resistance among pathogenic organisms is responsible for the rise of many life threatening diseases. Marine bacteria produce BS under fluctuating oceanic conditions such as oil-contaminated waters as well as secondary metabolites for their defence and survival against other micro-organisms. BS derived from the marine environment have been reported to inhibit cell adhesion and biofilm formation (Das et al. 2009a; Kiran et al. 2010a). They effectively show antimicrobial, anti-adhesive and biofilm disrupting activities against pathogenic micro-organisms. In addition, some have been found to display anti-tumour/anti-cancer activity and, as a result can be a potential source for future drugs (Table 2). Marine derived glycolipid BS show potential for the development of novel antibiofilm drugs. For instance, the marine actinobacterium *Brevibacterium casei* MSA19 produces glycolipid BS which significantly disrupts the biofilm formation in both mixed culture and individual strains at 30 μg glycolipid/ml (Kiran et al. 2010a). Another glycolipid BS from a marine *Staphylococcus saprophyticus* SBPS 15 showed antimicrobial activity against different human pathogenic clinical isolates. The BS was stable at a broad range of pH (3–9) and temperature (up to 80 °C) (Mani et al. 2016).

**Table 2:** Summary of biosurfactant/bioemulsifier suggested for biomedical applications produced by different marine microorganisms

Organism	Organism identification method(s)	Biosurfactant type	Biosurfactant property	BS/BE yield	Characterisation method	Reference
*Brevibacterium casei* MSA19	Morphological, biochemical and phylogenetic analysis	Glycolipid	Anti-biofilm, nanoparticle formation	18 g/l	Orcinol, TLC, FTIR, HPLC, GC–MS	Kiran et al. (2010a, 2010b)
*Staphylococcus saprophyticus* SBPS 15	16S rRNA	Glycolipid	Antimicrobial	1.345 ± 0.06 g/l	TLC, FTIR, MALDI-ToF–MS	Mani et al. (2016)
*B. circulans* DMS-2	Identified at MTCC	Lipopeptide	Anti-tumor	1.64 ± 0.1	HPTLC, FTIR, MALDI-ToF	Sivapathasekaran et al. (2010)
*Bacillus circulans*	Biochemical microbial identification method	Lipopeptide	Antimicrobial, anti-adhesion	1 g/l	UV–visible spectroscopy, TLC, HPLC, FTIR, MALDI-ToF	Das et al. (2009a), Mukherjee et al. (2009), Sivapathasekaran et al. (2009)
*B. subtilis* SDNS	16 s rRNA	*ε*-poly-l-lysine	Anti-cancer	76.3 mg/l	HPLC, TLC	El-Sersy et al. (2012)
*B. mojavensis* B0621A	16S rRNA	Lipopeptide	Cytotoxic activity, antifungal	15.5 g/24 l	HPLC, NMR, RP-HPLC, GC–MS	Ma and Hu (2014), Ma et al. (2012)
*Bacillus megaterium*	Morphological, physiological and biochemical tests	Lipopeptide	Anti-cancer	NA	RP-HPLC	Dey et al. (2015)
*Aneurinibacillus aneurinilyticus* SBP-11	16S rRNA	Lipopeptide	Antimicrobial activity and crude oil recovery	NA	TLC, GC–MS, MALDI-TOF–MS, FT-IR, NMR	Balan et al. (2017)
*Brevibacillus laterosporus* PNG-276	16S rRNA	Lipopeptide	Antimicrobial	NA	HPLC, MS, NMR	Desjardine et al. (2007)
*Staphylococcus lentus*	16S rRNA	Glycolipid	Anti-adhesive, anti-biofilm	NA	Orcinol, GC–MS, FTIR, TLC, HRMS	Hamza et al. (2017)

*NA* not available, *TLC* thin-layer chromatography, *FTIR* Fourier transform infrared spectrometry, *NMR* nuclear magnetic resonance, *HPLC* high performance liquid chromatography, *RP-HPLC* reversed phase-high performance liquid chromatography, *MALDI-ToF MS* matrix assisted laser desorption ionization-time of flight mass spectrometry, *GC* gas chromatography, *HPTLC* high performance thin layer chromatography, *HRMS* high-resolution mass spectrometer, *MTCC* microbial type culture collection, India

The biological activities of lipopeptides have been reported in marine bacteria. Marine derived lipopeptide, surfactin from *B. circulans* DMS-2 has potential antitumor activity against cancer cell lines HCT-15 (IC_50_ 80 μg ml^−1^) and HT-29 (IC_50_ 120 μg ml^−1^) (Sivapathasekaran et al. 2010). The lipopeptide BS produced by the marine *B. circulans* has anti-adhesive activity against several potential pathogenic strains. It was seen that BS-mediated surface conditioning significantly decreased bacterial adhesion of pathogenic strains like *E. coli*, *Micrococcus flavus* and *Proteus vulgaris* up to 89% at a concentration as low as 0.1 g/l showing its potential in biomedical applications (Das et al. 2009a). In another study a marine-derived *B. subtilis* SDNS produced *ε*-poly-l-lysine (*ε*-PL) which showed anti-cancer activity against human Hela S3 cell line (El-Sersy et al. 2012).

The molecular structure of a particular lipopeptide variant defines its biological activity. For example, the length of carbon-chain affects the antifungal activity in iturinic lipopeptides. Due to their hydrophobic nature the longer fatty acid chain interact effectively with the cell membrane. As reported, among three different lipopeptides isolated from marine derived *B. mojavensis*, the antifungal activity of fengycins (C_16_ and C_17_) were stronger than mojavensin A (C_15_) (Ma and Hu 2014; Ma et al. 2012). Likewise, the longer fatty acid chain isoform C_16_ of iturin A from marine *Bacillus megaterium* inhibited the proliferation of tumour cells by disrupting the Akt pathway leading to apoptosis (Dey et al. 2015). In another report, fengycin fractions produced by *B. circulans* strain, where out of four different fractions, antimicrobial activity was observed only with variants of C_16_ and C_17_ (Sivapathasekaran et al. 2009). The longer fatty acid chain of BS may offer advantages in increasing the surface-activity of these molecules.

These properties suggest that marine-derived BS can serve as a source of new biomolecules for the discovery of novel drugs. Other possible applications of BS can be the synthesis of metallic nanoparticles using marine-derived surfactants. Metallic nanoparticles are receiving great interest in the field of biomedicine for example, tissue engineering, drug delivery, detection of pathogens, detection of tumours, biological markers etc. (Salata 2004). Recently BS have been used both in nanoparticle synthesis and stabilization (Plaza et al. 2014). Microorganisms have developed the capability to grow and survive at high metal concentrations by various mechanisms such as, impermeable cell membrane, efflux of toxic ions, oxidation or reduction of ions and production of EPS (Decho 1990). Marine bacteria can bind a wide range of heavy metals through production of BS and contribute to organic carbon cycling (Das et al. 2009b). Therefore, utilization of marine derived BS is a safer route for environmental friendly synthesis of nanoparticles. In a study, a glycolipid BS was synthesized from sponge-associated marine *Brevibacterium casei* MSA19 under solid state fermentation. The glycolipid helped in stabilization of nanoparticles and prevented aggregation (Kiran et al. 2010b). Biosynthesis of metallic nanoparticles using bacteria and fungi has gained more attention while, synthesis of metallic nanoparticles using marine bacteria is an area yet to be fully explored (Plaza et al. 2014).

## Marine-derived BS in food and cosmetic formulations

Due to increased use of stabilizing and thickening agents in food products, these industries are looking for ingredients which can improve food quality and properties. Gum arabic, xanthan gum and lecithin are widely used hydrocolloid emulsifiers which efficiently emulsify and stabilize oil-in-water emulsions. In Quorn products (quorn.co.uk), dried fungus culture from *Fusarium venenatum* is used together with egg albumen as a binder. While, in vegan food products, potato protein is added in place of egg albumen. However, climate change and weather fluctuations such as drought will produce defoliation and affect the production of plant based gums. In order, to reduce the dependency on synthetic emulsifiers and plant based emulsifiers there is increased interest in finding new ingredients. Contrary to chemical surfactants, marine derived BS are non-toxic and/or less toxic with high stability at extreme temperature, pH and salinity.

Not many studies on the applications of marine-derived BS in food and cosmetic formulations have been reported so far. The only commercialized microbial emulsifier is Emulsan produced from *A. calcoaceticus*. BS, which due to its emulsion stabilizing property, can improve consistency, texture and solubilisation of fat globules, and aroma in food products. Incorporation of BE has been found to improve the rheology of dough, increasing the volume and emulsification of fat and thus finds useful applications in the bakery and meat processing industries. For example, a glycoprotein emulsion stabilizer produced by marine *Antarctobacter* sp. TG22 can produce stable oil-in-water emulsions with commercial food-grade oils (Gutierrez et al. 2007a). Similarly two other glycoprotein emulsifiers from marine *Halomonas* species TG39 and TG67 show superior emulsifying properties when compared to commercial emulsifiers with stable activity under acidic conditions and high temperatures (Gutierrez et al. 2007b).

The lipopeptide MSA31 from a marine *Nesterenkonia* species is an effective emulsifier, with good antioxidant activity and its addition to muffins improved softness and retained food quality. The lipopeptide MSA31 effectively reduced 90% of biofilm formed by *Staphylococcus aureus* and was non-toxic to the brine shrimp nauplii (up to 200 µg/ml) (Kiran et al. 2017). Another bioemulsifier produced by a marine bacterium *Enterobacter cloacae* was reported to promote the viscosity of acidic food products (Iyer et al. 2006). In the food industry, there is wide scope for marine derived-BS as emulsifiers, stabilizing agents, antimicrobial and antiadhesives/antibiofilm agents (Table 3).

**Table 3:** Summary of biosurfactant/bioemulsifier suggested for food/cosmetic applications produced by different marine microorganisms

Organism	Organism identification method(s)	Biosurfactant type	Biosurfactant property	Biosurfactant yield	Characterisation method	Reference
*Antarctobacter* sp. TG22	16S rRNA	Glycoprotein	Emulsion-stabilizing agent	21.1 mg/l	HPLC, GC, Size-exclusion chromatography, NMR	Gutierrez et al. (2007a)
*Halomonas* species TG39 and TG67	16S rRNA	Glycoprotein	Emulsification and stabilisation of food oils	131.0 ± 0.07 mg/l, 28.0 ± 0.01 mg/l	HPLC, NMR, GC	Gutierrez et al. (2007b)
*Nesterenkonia* species MSA31	16S rRNA	Lipopeptide	Emulsifier-stabilizing agent in food, anti-biofilm, antioxidant	NA	GC–MS, FTIR, TLC, NMR	Kiran et al. (2017)
*Enterobacter cloacae*	API system	EPS	Emulsifier-stabilizing agent in food	NA	NA	Iyer et al. (2006)
*Nocardiopsis* VITSISB	NA	NA	Emulsifying and foaming agent in toothpaste	NA	NA	Das et al. (2013)
*Pseudomonas fluorescens* BD5	16S rRNA	Lipopeptide	Anti-melanoma compound	10 mg/l	RP-HPLC, MALDI-ToF–MS, MS/MS	Janek et al. (2010, 2013)

*NA* not available, *API* analytical profile index, *TLC* thin-layer chromatography, *FTIR* Fourier transform infrared spectrometry, *NMR* nuclear magnetic resonance, *MS/MS* tandem mass spectrometry, *RP-HPLC* reversed phase-high performance liquid chromatography, *MALDI-TOF MS* matrix assisted laser desorption ionization-time of flight mass spectrometry, *GC* gas chromatography

Chemicals used in cosmetic formulations often cause skin irritations and allergies. There is significant interest in natural cosmetic products among consumers. Replacement of SACs in these products with BS can reduce such harmful effects. The surface-active properties are essential to determine the type and amount of BS in detergents, cosmetic, pharmaceuticals and various other industries. The type of BS compound to be incorporated in the formulations can be selected based on their emulsifying ability and/or surface-activity such as, hydrophilic–lipophilic balance (HLB) and critical micelle concentration (CMC), respectively. The HLB defines the polarity of the BS, which gives an indication of its solubility in different systems. BS with a high HLB value indicates that it is highly hydrophilic while, a low HLB value shows a high lipophilic character. Based on the HLB values, a BS will be an emulsifier, antifoaming agent and wetting agent which are desirable properties in cosmetic products.

CMC is the minimum concentration of BS required to lower the ST of water. At the CMC surfactant molecules form micelles to reduce surface tension and interfacial tension. The surface-active properties of a BS are determined by its side chain length, unsaturated bonds and the size of hydrophilic group. With increasing hydrophobicity, the CMC of BS molecules tends to decrease. That means, a lower concentration of BS is required for micelle formation. It is reported, *Burkholderia thailandensis* producing rhamnolipids Rha–Rha–C_14_–C_14_ has a CMC of 225 mg l^−1^ compared to *P.* *aeruginosa* PG201 producing Rha–Rha–C_10_–C_10_ of 600 mg/l. Due to the hydrophobicity of the longer fatty acid chains, *Burkholderia* BS has a lower CMC compared to *P. aeruginosa* (Dubeau et al. 2009).

Considering the foaming and emulsifying properties, BS can be used for different applications in health care products including, cleansers, moisturizers, toothpaste and personal care products. Marine-derived BS effectively show antimicrobial, anti-adhesive and biofilm disrupting activities against pathogenic microorganisms. This property can be utilised in cosmetic and skin care products. Similar to skin cell membranes, the fatty acid chain in BS can prevent generation of free radicals from UV radiation. Therefore, BS can be applied as antioxidants in skin care products (Vecino et al. 2017).

Before introducing BS in industrial formulations, it is necessary to determine the toxicity of these SACs on cells or animal models. For example, the toxicity of glycolipid BS (BS-SLSZ2) produced by a marine epizootic bacterium *Staphylococcus lentus* towards eukaryotic model organism was determined. The glycolipid BS-SLSZ2 efficiently inhibited biofilm formation in *Vibrio harveyi* and *P. aeruginosa.* In vivo experiments showed that BS-SLSZ2 was non-toxic towards *Artemia salina* and was effective in protecting *A. salina* against *V. harveyi* and *P. aeruginosa* infections (Hamza et al. 2017). In another report, marine *Pseudomonas* sp. MK90e8 and MK91CC8 was reported to produce massetolide A, novel cyclic depsipeptide and viscosin, repsectively. Massetolide A and viscosin exhibited in vitro antimicrobial activity against *Mycobacterium tuberculosis* and *Mycobacterium avium*-*intracellulare.* The effect of massetolide A was found to be non-toxic to mice at a dose of 10 mg/kg thus showing potential in treating infections against *Mycobacterium* (Gerard et al. 1997).

The activity of Pseudofactin II (PFII), a lipopeptide BS isolated from the Arctic strain of *P. fluorescens* BD5 was compared with normal human dermal fibroblast (NHDF). PFII induced apoptosis of melanoma skin cancer cells while NHDF were less affected under same conditions. The mechanism of melanoma cell death may be due to increased plasma membrane permeability by BS micelles. The activity of PFII was most effective above CMC (130–140 µM) (Janek et al. 2013). The toxicity of BS from marine bacteria *Nocardiopsis* VITSISB was evaluated in toothpaste formulation. BS was found to more efficient and less toxic surfactant compared to chemical surfactant sodium lauryl sulphate (Das et al. 2013). Further investigations should be done to supply safe and effective products to satisfy consumers’ demands.

## Recommendation and conclusion

Due to their interesting biological properties, BS from extremophiles have great potentials with a broad range of applications for industrial and consumer products. Considering their properties such as, emulsifiers, thickeners, anti-oxidants, extreme tolerance to pH or temperature as well as antifungal and antimicrobial activities we look forward to potentially new products with marine-derived BS as their surface-active ingredients. Although highly desirable they also have their disadvantages such as the production of relatively low yield of BS and high production costs. In some studies, either the product yield is very low or have not been quantified. Additionally, in some studies, the taxonomic identification of strains has not been carried out using reliable methods. As suggested by Irorere et al. (2017) criteria should be followed before claims for a BS producing strain and the identity of the product are made. In comparison to BS produced from mesophilic microorganisms, not much work has been done on marine BS. Fermentation optimisation by varying physiochemical factors including, temperature, pH, aeration and agitation speed and nitrogen would be beneficial to increase the product yield. In order to develop robust BS producing strains it is important to identify the genes involved in SACs biosynthesis and to investigate the regulatory mechanisms involved in BS synthesis by targeting those regulatory genes.

Close examination of the information presented in Tables 1, 2 and 3 reveals the deficiencies in the data currently available from the published literature. Although many of the organisms have been reliably identified using 16S rRNA sequence data, other identifications have relied on morphological, physiological and biochemical tests. This lack of specific taxonomic data makes extrapolations from one study to another significantly more difficult. The other glaring lack of information concerns the yield of BS produced either as a crude yield or as a purified material. As we have already pointed out one important issue dictating whether BS will become a common constituent of consumer products or will be used in bulk bioremediations is cost. We can see from the few yield figures given that in general they are exceptionally low, of the order of mg/l, rather than g/l. In the case of one microbial biosurfactant that has reached industrial use, the sophorolipid from *Starmerella bombicola*, yields are of the order of hundreds of g/l. Until the production from marine organisms can be increased, either through the manipulation of the growth conditions or through genetic manipulation, there is little prospect of major commercial interest.

The final point we can take from data in the tables is the lack of consistency and reliability of the methods used to identify the products. In some instances, no real attempts have been made to specifically identify the products and in others general analytical methods have been used which only provide indicative information concerning the structures. In many of the applications under considered for BS, particularly by the pharmaceutical and cosmetic industries, specific knowledge of the compounds being produced is imperative. In many cases microbial BS are typically a mixture of different congeners and where bioactivity is being investigated it is critical that pure samples of individual congeners are used in order to be able to assign a specific bioactivity to a molecule. Mixtures of different molecules may have competing activities which may cancel each other out.

Some studies have been done on potential rhamnolipid producing strains including *P. aeruginosa* and *Burkholderia* species to enhance rhamnolipid production. One such approach is cloning the rhamnolipid biosynthesis genes *rhlA* and *rhlB* into non-rhamnolipid producing strains, *E. coli* BL21 (DE3) and *P. aeruginosa* PAO1 *ΔrhlA*. However, the BS yield from the recombinant strains was too low in comparison to the wild type strains (Wang et al. 2007). Another approach to increase the BS yield is to silence the genes competing with the BS production pathway. For example, Funston et al. (2017) knocked-out the polyhydroxyalkanoate (PHA) pathway synthesis genes for enhanced rhamnolipid production. The halophile *Halomonas* species are ubiquitous in marine environments and produces BE which effectively emulsifies hydrocarbons and other petroleum contaminants. These species are known BS producers as well as PHA producers. However, the effect of PHA deficient mutation on the yield of its SACs is not known. It might be interesting to follow a similar strategy in *Halomonas* species to reduce the carbon flux towards other metabolic pathways and increase the BS production.

The chemical composition and toxicity must be known before commercialisation of the SACs. The developed techniques such as high-performance liquid chromatography (HPLC) and ultra-performance liquid chromatography tandem mass spectrometry (UPLC-MS/MS) can be used to analyse BS congeners (Rudden et al. 2015). The analysis and purification of a particular BS fraction displaying bioactivity is a prerequisite for subsequent biomedical applications. Reverse-phase high-performance liquid chromatography (RP-HPLC) has recently been used to fractionate and purify glycolipid and lipopeptide compounds (Sivapathasekaran et al. 2009). The separation and purification of BS can enhance the level of bioactivity of BS isoforms. The antimicrobial activity of the lipopeptide was reported to increase further after purification (Mukherjee et al. 2009). While, the toxicity of BS can be tested in eukaryotic models such as, *Caenorhabditis elegans* and *Artemia* sp. The toxicity of the BS producing strain can be tested in the *Galleria mellonella* model. The Galleria model was used to test the pathogenicity in rhamnolipid BS producing strains and it was found that *B. thailandensis* was significantly less pathogenic than *P. aeruginosa* (Irorere et al. 2018) and that a marine Pseudomonad was also non-pathogenic (Twigg et al. 2018).

Marine-derived BS effectively emulsify hydrocarbons therefore, are suitable for marine-based bioremediation. The improved biodegradation levels obtained with marine-derived BS indicate that they represent the most efficient accelerators for hydrocarbon biodegradation through increasing the bioavailability of oil. Use of extremophiles such as, *Acinetobacter*, *Halomonas*, *Marinobacter* which effectively produce BS and participate in marine bioremediation should be preferred over mesophilic microorganisms for remediation of oil spills. Psychrophilic bacteria are capable of degrading crude oil efficiently at low temperatures. Therefore, BS produced from psychrophilic bacteria are suitable for bioremediation of hydrocarbon contaminated sites in cold environments. For bioremediation purposes, the impurities within the BS mixture act as a co-substrate and enhance the degradation of pollutants (Mata-Sandoval et al. 2001). Considering the downstream processing costs further purification of BS is not required for bioremediation purposes.
